# Everybody's Page

**Published:** 1892-03-12

**Authors:** 


### EVERYBODY'S PAGE.

OWING to the introduction of tuberculin a marked stimulus has been given to hypodermic medication. M. Darier, a French surgeon, is a notable exponent of this method in the treatment of affections of the eye. In cases of optic nerve failure, the action of strychnia is asserted to be decidedly more efficacious if injected in the region of the orbit.

HOT-AIR baths, so writes Dr. Paul Chevandier, were introduced into Europe by the Moors—one of the few good things we have to thank these people for. The same authority states that at the time of the discovery of America, the natives of Mexico used heated air saturated with aromatic vapours for the treatment of rheumatism. Their method of procedure was to dig a hole in the ground long enough to lie down in, which was roofed over with a dome of clay, and the floor of the whole was strewn with aromatic shrubs.

A CURIOUS and unpleasant case of poisoning happened some time ago to a foreign actress. When the doctor was summoned the victim was foaming at the mouth, her face was pallid, her extremities cold, the pulse was intermittent, and the respiration superficial and slow. Having decided that the cause of this condition was the inhalation of illuminating gas, he used injections of ether. As these proved abortive the doctor resorted to nitro-glycerine, of which he injected 1-64th grain into the prœcordial region, the result was prompt improvement and rapid recovery of the patient.

ACCORDING to a German expert, Dr. Kolb, who has been making experiments with a delicate machine for measuring the beats of the pulse, cycling is much less exhausting to ordinary persons than running, which, he alleges, is the most exhausting of all physical exercises. How about rowing? Seeing that there is greater tax on the "wind" of a runner than upon that of a cyclist, this does not seem an extraordinary discovery. Still it is satisfactory to be told that weakness in the action of the heart or arteries seldom results from cycling.

THE time has come round again for the annual depredation of plovers' nests. The cruelty of this plundering, which takes place on such a large scale, does not seem to strike those who are ordinarily imbued with feelings of kindness for birds and animals in general. The case is one in which the wrong is done by the wealthy, who ought to set a better example to their poorer brethren. It is one thing for a rich man to rob a bird's nest for the gratification of his appetite, another for a costermonger to illtreat his donkey. Till people refuse to buy plovers' eggs at extravagant prices the wrong will continue.

IT appears as though little would be left soon to private enterprise in Germany. The proposal that the preparing and dispensing of drugs throughout the Empire should be taken over by the State is not a novel one, and if persisted in will in all probability become an accomplished fact eventually in a country where officialism flourishes with such vigour.

PROFESSOR CRAFTS, of Boston, U. S. A., has thoughtfully discovered for long-suffering humanity a new source of danger. In London we are only too well acquainted with the deleterious effects of the sulphur which is generated from every ton of coal burnt, but the Professor points out that in London, where coal is burnt so freely, a large quantity of arsenious acid is given out into the air and also lodges in the chimneys. So if we get rid of the bug-bear of arsenical poisoning from wall papers we still have our enemy with us in coal, and are literally out of the frying pan into the fire.

A FRENCH newspaper tells a touching story of a patient's gratitude to her physician for his intelligent and devoted care of her health. This grateful lady died at the ripe age of eighty-three, and in order to testify to the value in which she held her doctor's services she left to him in her will everything contained in her private cabinet. On opening this casket of treasures, the worthy doctor discovered, no doubt to his unbounded satisfaction, that its only contents were boxes of pills and untouched mixtures which he had prescribed during the past ten years for the deceased.

ANTI-VIVISECTIONISTS must have gained heart and renewed hope for the ultimate success of their cause in the marked differences of opinion among physiologists upon the action of chloroform, and what is unsatisfactory to the physiologist is a matter of satisfaction to the anti-vivisectionist. In addition to the Glasgow Committee on Anæsthetics in 1879 and the Hyderabad Commission, which considered amongst other questions the comparative fatality of ether and chloroform and the mode in which they kill—whether through the circulation or the respiration—a series of experiments has more recently been made by Professor Macwilliam, of Aberdeen, and Professor Wood, of Pennsylvania, which seems to make confusion worse confounded. The safest and most practical conclusion to draw from the remarkable differences obtained in results and opinions seems to be, that experiments on animals are of little if any avail for a solution of the vexed questions. Even if clinical observation is as unsatisfactory in its results, it has not the great objections against it possessed by experiments on living animals.

